# Endothelial Dysfunction in Heart Failure: What Is Its Role?

**DOI:** 10.3390/jcm13092534

**Published:** 2024-04-25

**Authors:** Andrea Drera, Luca Rodella, Elisa Brangi, Mauro Riccardi, Enrico Vizzardi

**Affiliations:** 1 Institute of Cardiology, ASST Spedali Civili di Brescia, Department of Medical and Surgical Specialties, Radiological Sciences, and Public Health, University of Brescia, 25123 Brescia, Italy; a.drera002@unibs.it (A.D.); l.rodella004@unibs.it (L.R.); e.brangi@unibs.it (E.B.); mauro94rc@hotmail.it (M.R.); 2 Cardiology Unit, Department of Medical and Surgical Specialties, Radiological Sciences and Public Health, Spedali Civili di Brescia, 23123 Brescia, Italy

**Keywords:** heart failure, endothelium, endothelial dysfunction, nitric oxide, inflammation

## Abstract

The endothelium is a continuous layer of cells that coats the interior walls of arteries, capillaries, and veins. It has an essential regulatory role in hemostatic function, vascular tone, inflammation, and platelet activity. Endothelial dysfunction is characterized by a shift to a proinflammatory and prothrombic state, and it could have a bidirectional relationship with heart failure (HF). Due to neurohormonal activation and shear stress, HFrEF may promote endothelial dysfunction, increase ROS synthesis, and reduce nitric oxide production. Different studies have also shown that endothelium function is damaged in HFpEF because of a systemic inflammatory state. Some clinical trials suggest that drugs that have an effect on endothelial dysfunction in patients with HF or cardiovascular disease may be a therapeutic option. The aim of this review is to highlight the pathogenetic correlation between endothelial dysfunction and heart failure and the related potential therapeutic options.

## 1. Endothelial Functions

The endothelium is a continuous layer of cells lining the blood vessels [1]. It has an essential regulatory role in hemostatic function, vascular tone, inflammation, and platelet activity [2].

Vascular tone regulation is an important endothelium function; under physiological conditions, homeostasis is maintained in favor of vasodilation by controlled synthesis and the release of endothelium-derived relaxing factors [3]. In fact, the shear stress generated by flowing blood and the resulting force are transduced into biochemical signals that regulate homeostasis by producing different molecules [4], such as prostaglandins, endothelium-dependent hyperpolarization factors, carbon monoxide, arachidonic acid metabolites, hydrogen peroxide [5], and nitric oxide [6]. Nitric oxide is an important mediator and has numerous molecular targets and functions. It is generated by three isoforms of nitric oxide synthase: neuronal, inducible, and endothelial NOS, respectively [7]. Endothelial nitric oxide synthase, which is mostly expressed in endothelial cells, induces cGMP-dependent vasodilatation through the activation of guanylate cyclase [8]. Nitric oxide is also an inhibitor of platelet aggregation and adhesion to the vascular wall, and it controls the platelet-derived growth factor release that stimulates smooth muscle proliferation and matrix molecule production [7].

In addition to the shear stress and the resulting mechanical force, many vasoactive mediators, such as acetylcholine, histamine, serotonin, thrombin norepinephrine, bradykinin, isoproterenol, and substance P, can also stimulate NO production [9].

The endothelium also regulates hemostasis and thrombosis, providing an interface between tissue and blood [2]. A complex balance exists between the pro- and anticoagulant systems regulated by the endothelium: in the quiescent state, endothelial cells promote the activity of numerous anticoagulant pathways; however, if activated by different stimuli, e.g., cytokines, they shift to a procoagulant state [10].

The endothelium also regulates inflammation [11] by producing antiproliferative and anti-inflammatory cytokines [12], and it plays an important role as a physical barrier in blood circulation [10].

## 2. Methods for Measuring Endothelial Function

There are various methods for measuring endothelial function, such as intracoronary arterial infusion of vasoactive substances, like acetylcholine (Ach), in addition to quantitative coronary angiography (QCA), with or without Doppler imaging, followed by flow-mediated vasodilation (FMD) and peripheral arterial tonometry (PAT).

Intracoronary Ach infusion was the first method that was discovered for measuring endothelial function and is the most reliable; however, because it was also the most invasive method, ACh was almost completely replaced by FMD. However, FMD and QCA with acetylcholine both require skilled technicians; so, a new and less invasive method, known as PAT, is now emerging and is under investigation prior to validation as a possible FMD replacement.

### 2.1. Intracoronary Artery Infusions Using Acetylcholine or Other Vasoactive Substances

In 1986, the introduction of acetylcholine infusion during quantitative coronary angiography (QCA) marked the pioneering approach for assessing endothelial function, and it is now widely acknowledged as the gold standard for early dysfunction detection [13,14].

The intracoronary arterial infusion stands out as the most direct, invasive, and costly method employed to gauge endothelial function. Within the QCA procedure, an iodate-contrast agent is intricately injected into the coronary arteries; thus, it is possible to directly see the vessels and to measure the diameters of numerous projections. After obtaining baseline diameters, ACh is infused into the arteries, and using Doppler techniques, it is possible to continuously measure coronary blood flow. In contrast to the QCA procedure, intravascular ultrasound (IVUS) can be utilized with Ach or other vasoactive infusions to evaluate coronary blood flow velocity, using a Doppler-guided wire to obtain vessel measurements. Regardless of whether quantitative coronary angiography (QCA) or intravascular ultrasound (IVUS) is employed, evaluating endothelial function involves the computation of volumetric coronary blood flow and its analysis on a dose-response curve [15]. In a normally functioning endothelium, the administration of acetylcholine (Ach) leads to coronary artery dilation. Conversely, in patients with endothelial dysfunction, Ach elicits a paradoxical vasoconstriction of the arteries [14]. Both QCA and IVUS are extensively validated techniques and have demonstrated their usefulness in the study of endothelial function among individuals with cardiovascular disease and heart failure [16,17].

Schächinger et al. [16] found that the progression of atherosclerotic disease and the risk of cardiovascular events in the long term could be predicted by coronary endothelial vasodilation dysfunction. In a recent study by Reriani et al. [18], it was highlighted that coronary endothelial function can serve as a valuable tool for the reclassification of the risk stratification of individuals with early coronary artery disease (CAD). Nevertheless, due to the invasive nature of the procedure and the elevated risks linked to coronary catheterization, conducting intracoronary studies becomes impractical when screening large populations (Table 1).

### 2.2. Flow-Mediated Vasodilation (FMD)

Introduced in 1992, flow-mediated vasodilation (FMD) has become widely accepted as a noninvasive method for assessing endothelial dysfunction, and it is recognized for its robustness and validation [19]. To conduct FMD measurements, the patient assumes a supine position, and a blood pressure cuff is placed above the antecubital fossa. After capturing a baseline image of the brachial radial artery, the cuff is inflated to 40 to 50 mmHg above the systolic pressure, temporarily obstructing arterial inflow for a brief period. Upon cuff deflation, a substantial shear stress occurs, allowing induced reactive hyperemia to dilate the brachial artery. Nitric oxide (NO), released from endothelial cells and other vasoactive mediators, is responsible for inducing this shear stress. The ultrasound continuously records changes in the brachial radial artery’s diameter for up to 2 min after cuff deflation, and FMD is quantified as a percentage change in the post-stimulus diameter compared to the baseline diameter.

Flow-mediated vasodilation is favored in clinical trials due to its user-friendly nature, efficiency, and noninvasiveness. However, various factors such as food intake, coffee consumption, drugs, vitamins, exercise, tobacco use, ambient temperature, and the menstrual cycle can impact a patient’s FMD, potentially introducing confounding elements into the study results.

Hence, it is crucial for patients to undergo fasting prior to the assessment and to refrain from taking vitamins and medications, engaging in physical activity, and using tobacco on the test day. Additionally, maintaining consistent conditions in the study room throughout the trial is paramount. This involves ensuring a stable and comfortable room temperature, consistent lighting, and minimal environmental stress. Numerous cardiovascular studies employing flow-mediated dilation (FMD) have provided valuable results on the role of endothelial dysfunction in CVD and heart failure. It has been observed that flow-mediated vasodilation is significantly compromised in individuals with conditions such as hypertension, atherosclerosis, ischemic cardiomyopathy, coronary artery disease, dilated cardiomyopathy, congestive HF, and peripheral artery disease [20,21]. Furthermore, the research utilizing FMD indicates its significance as a valuable prognostic tool for diagnosing CVD [22] (Table 1).

### 2.3. Peripheral Arterial Tone (PAT)

With the growing acknowledgment of endothelial dysfunction’s role in heart failure (HF) and the crucial predictive function of accurate assessments in detecting cardiovascular events, there arises a need for innovative noninvasive techniques. Peripheral arterial tone (PAT) emerged in the early 2000s as a novel noninvasive technique to assess peripheral microvascular endothelial dysfunction. The concept of gauging peripheral microvascular endothelial dysfunction emerged, leading to the introduction of EndoPAT as a quicker, more cost-effective, and simpler method for evaluating endothelial function.

EndoPAT utilizes the peripheral arterial circulation in the finger. In a similar manner to brachial flow-mediated dilation (FMD), hyperemia is induced by temporarily obstructing blood flow through the brachial artery using a cuff for a duration of 5 min. The device subsequently assesses alterations in arterial tone in peripheral arterial beds, usually in the index finger. Following cuff release, the device autonomously computes reactive hyperemia as a ratio of post-occlusion to pre-occlusion signals, thereby eliminating operator dependence. Additionally, a baseline measurement is taken in the other arm, which serves as an internal control. A significant limitation of PAT is the unclear physiology. Unlike FMD, which assesses macrovascular dilatation, PAT measures microvessel dilatation, partially by relying on nitric oxide (NO). Interestingly, the inhibition of nitric oxide (NO) and the subsequent assessment of endothelial function through peripheral arterial tonometry (PAT) results in a reduction in the response of less than 50%. In contrast, when utilizing flow-mediated dilation (FMD), the response is nearly completely inhibited [23]. Although both technologies are designed to gauge endothelial function, various studies aiming to validate PAT by establishing correlations with FMD have yielded mixed results [24]. Hamburg et al. [25] and Lee et al. [26] discovered no correlation, raising doubts about the reliability of PAT. Conversely, Kuvin et al. [27] identified a notable correlation between reactive hyperemia measured by flow-mediated dilation (FMD) and that measured by peripheral arterial tonometry (PAT). Despite the diverse findings when comparing FMD and PAT, PAT has been acknowledged as a viable diagnostic tool for patients with heart failure (HF) [28,29] (Table 1).

## 3. Endothelial Dysfunction

Endothelial dysfunction is characterized by a shift in the actions of the endothelium and encompasses various nonadaptive modifications in the functional phenotype, which results in alterations in the regulation of hemostasis and thrombosis, vascular tone and redox balance, and inflammatory dysregulation [30].

**Figure 1:**
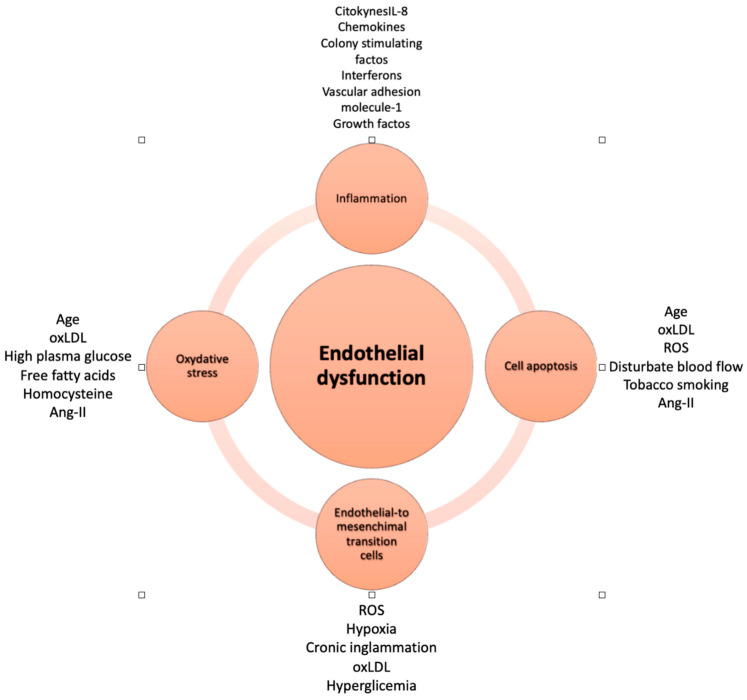
Endothelial dysfunction mechanisms (ROS: reactive oxygen species, Ang II: angiotensin-2).

The pathophysiology is complex and involves multiple mechanisms (Figure 1).

Oxidative stress is an important factor in endothelial dysfunction pathogenesis. It is derived from various enzymatic pathways, such as xanthine oxidase, NADPH oxidases, uncoupled eNOS, and dysfunctional mitochondria and occurs when the balance between pro-oxidant and antioxidant systems breaks up [2,31].

Excessive ROS levels oxidize intracellular macromolecules and lead to a reduction in the production of NO by inducing the formation of peroxynitrite [31], a cytotoxic oxidant that leads to degradation of the eNOS cofactor tetrahydrobiopterin [8], resulting in the “uncoupling” of eNOS and pro-oxidant activity. Oxidative excess is also linked to reduced endothelial vasodilatation and a proinflammatory state. Finally, it also upregulates the expression of adhesion molecules, e.g., ICAM-1 and VCAM-1, and chemotactic molecules [31].

Inflammation plays an important role in the pathogenesis of cardiovascular disease [32]. In response to injury, endothelial cells produce different molecules (chemokines, interleukin-8, colony-stimulating factors, interferons, monocyte chemoattractant protein-1, intercellular adhesion molecule-1, E-selectin, P-selectin, vascular adhesion molecule-1, growth factors, and other inflammatory factors) [2]. Consequently, it causes an increased adhesion and migration of leukocytes across the endothelium, activating a proinflammatory state [33]. Furthermore, proinflammatory mediators stimulate endothelial cells to secrete other proinflammatory cytokines, activating a vicious circle [34].

This alteration results in a proinflammatory, proliferative, and prothrombotic state characteristic of dysfunctional endothelial cells [35,36].

Moreover, endothelial dysfunction is also characterized by other alterations, such as increased cell apoptosis and endothelial-to-mesenchymal transition cells [37], leading to vascular leakage, inflammation, and coagulation.

## 4. Endothelial Dysfunction and Heart Failure

HF is a syndrome characterized by a structural and functional alteration of diastolic and/or systolic function. As the ESC Guidelines report, we can recognize HF with a preserved ejection fraction (HFpEF, LVEF > 50%) and a reduced ejection fraction (HFrEF, LVEF < 40%), as well as a third class with a mild systolic dysfunction (HFmrEF, LVEF 41–49%).

HFrEF could be the result of either ischemic (i.e., ACS) or nonischemic damage (i.e., myocarditis, Takotsubo syndrome, or a genetic cause, for example). The HFrEF standard of care is represented by four main classes of medications: Sacubitril/Valsartan (ARNI) or RAASi (ACE-i/ARBs), aldosterone receptor antagonists (MRA), beta-blockers, and SGLT2 inhibitors. These molecules allow reverse ventricular remodeling and an improvement in LVEF and have shown a significant reduction in cardiovascular death and in HF hospitalizations with an improvement in clinical outcomes [38].

In HFpEF, different comorbidities, such as hypertension, diabetes, chronic kidney disease, obesity, anemia, and lung diseases may be responsible for the etiology even without directly damaging the heart [38,39]. These comorbidities contribute to a systemic inflammatory state, which is also responsible for oxidative stress in the coronary microvascular endothelium. Myocardial remodeling in HFpEF is different from that in HFrEF, where it is secondary to cardiomyocyte death due to ischemia, infection, or toxicity. Only SGLT2-i demonstrated good results in terms of mortality reduction in this type of patients [40].

Endothelial dysfunction is involved in heart failure (HF), although their relationship remains unclear.

Some authors have reported that HFrEF could promote endothelial dysfunction because of neurohormonal activation and shear stress and could increase ROS synthesis (especially hydrogen peroxide and peroxynitrite) and reduce nitric oxide (NO) production [11]. The imbalance between NO and oxidative stress causes a decrease in endothelium-dependent vasodilation, which becomes particularly relevant in coronary vessels, where it leads to a reduction in myocardial perfusion and ventricular function. The continuation of this process can be responsible for the progression in chronic HF [11]. A study involving 24 patients with chronic heart failure (CHF) and systolic dysfunction (NYHA class II–III) analyzed the endothelium-dependent vasodilatation invasively via venous occlusion plethysmography (VOP) and demonstrated a reduction compared to 22 control subjects [41].

On the other hand, different studies have also shown endothelial damage in HFpEF, which is probably due to a systemic inflammatory state [42,43,44]. The production of cytokines, mainly TNF-alpha, is responsible for endothelial cell transformation into fibroblasts and for the down-regulation of eNOS expression and is therefore related to the degree of endothelial dysfunction [45,46]. This process is related to the reduction in cyclic-guanosine monophosphate (c-GMP) that is responsible for microvascular ischemia and increased diastolic cytosolic calcium and, consequently, altered myocardial relaxation [12,39]. NO imbalance also alters endothelial progenitor cells and therefore impairs endothelial repair [12].

Furthermore, the down-regulation of eNOS shifts the cardiac metabolism from free fatty acids to lactate, which reduces exercise tolerance, and it is responsible for endothelial cell apoptosis [11]. Matrix metalloproteinases become dysfunctional, causing the accumulation of extracellular matrix structural protein and consequently inducing cardiac hypertrophy, fibrosis, and myocardial stiffness, which ultimately leads to progressive diastolic dysfunction. The alteration of metalloproteinases can also reduce atherosclerotic plaque stability, resulting in an augmented ischemic risk [47,48].

It is now clear that left ventricular remodeling is also largely regulated by eNOS and NO signaling: mice lacking eNOS have worse left ventricular remodeling after myocardial infarction compared to wild types.

Because of this, it is vital to maintain adequate endothelial eNOS production and eNOS activity; endothelial hyporesponsiveness in the coronary circulation and reduced eNOS expression have been documented in an HF model of ventricular pacing in dogs [49].

**Figure 2:**
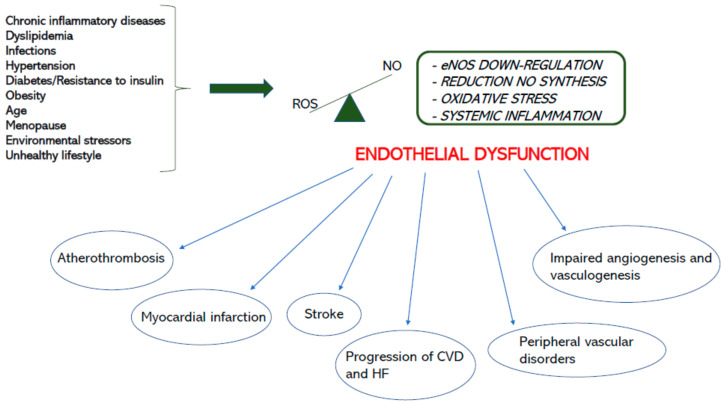
Endothelial dysfunction and CV consequences. (CVD: cardiovascular diseases, HF: heart failure, ROS: reactive oxygen species, NO: nitric oxide, eNOS: endothelial nitric oxide synthase).

**Figure 3:**
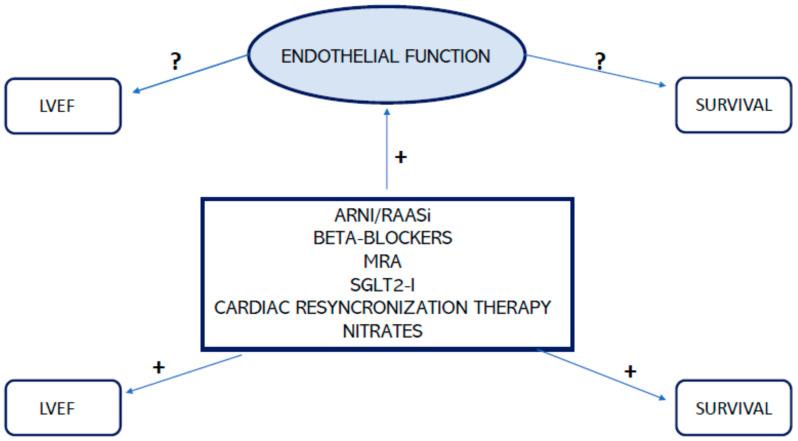
Effect of heart failure therapies on LVEF and endothelial function. (ARNI: angiotensin receptor nepylisin inhibitor; RAASi: renin–angiotensin–aldosterone system inhibitors; MRA: mineralcorticoid receptor antagonist; LVEF: left ventricular ejection fraction).

Finally, a dysfunctional endothelium is responsible for increased vascular stiffness and impaired arterial distensibility (Figure 2). Endothelin-1 production is increased in HF, causing smooth muscle cell growth and vascular remodeling [11]. The degree of endothelial dysfunction is related to HF severity and predicts adverse clinical events, including mortality. ACE inhibitors improve endothelial function by reducing oxidative stress and increasing bradykinin levels. These molecules can actively reduce vascular remodeling and atherosclerosis, improving arterial elasticity [50,51]. Carvedilol and Nebivolol have a vasodilating and antioxidant activity that allows better endothelial function, which decreases vascular stiffness [46,52] (Figure 3). Nitrates and PDE5 inhibitors increase NO availability, vasodilation, and myocardial contractility, and they reduce the ventricular afterload, which improves pulmonary hemodynamics [53,54,55]. Finally, statins have also demonstrated good results in the improvement of arterial distensibility and in reducing inflammation [16]. As stated above, HF treatments are less effective for HFpEF compared to HFrEF, but it is possible to assume that targeting endothelial activity could help to reduce CV events in the first group. Indeed, it has already been demonstrated that an improvement in endothelial function could be related to better outcomes.

## 5. Endothelial Dysfunction in Acute Heart Failure

The management of acute heart failure (AHF) has seen minimal change over the years and has relied on standard medications like loop diuretics, vasodilators, vasopressors, and inotropes [38,56]. AHF is a multifaceted clinical syndrome stemming from the sudden onset or exacerbation of existing cardiac dysfunction, impairing the heart’s ability to fill and pump blood effectively. This leads to symptoms of heart failure (HF), necessitating urgent admission to the emergency department and unplanned hospital stays.

AHF may be either the initial indication of HF (new onset) or, more commonly, the result of an acute worsening of chronic HF. In comparison to patients experiencing acute decompensation of chronic HF, those with new onset HF might face higher in-hospital mortality [57] rates, but they exhibit lower rates of mortality and rehospitalization after discharge. Various external factors may trigger, though not directly cause, AHF in individuals with pre-existing heart conditions [58,59,60].

The severity of the condition and its trajectory during hospitalization are influenced by the intricate interplay between triggers, underlying cardiac conditions, and the patient’s comorbidities.

In the 2021 ESC guidelines for heart failure diagnosis and treatment, four primary clinical presentations are outlined and may overlap. These presentations are primarily distinguished by the presence of congestion signs and/or peripheral hypoperfusion, necessitating different therapeutic approaches. The four presentations are acute decompensated heart failure (ADHF), which is the most prevalent form of AHF, acute pulmonary edema, right ventricular failure, and cardiogenic shock.

De novo acute heart failure (AHF) arises in individuals with no prior history of heart disease and apparently normal cardiac function. The progression towards irreversible cardiac damage in such cases depends on the interaction between various factors, including etiology (both ischemic and nonischemic), demographic variables (such as age and sex), the presence of comorbidities (e.g., diabetes, chronic kidney disease, anemia, chronic obstructive pulmonary disease, depression, and genetic predisposition), and the timing of pharmacological and nonpharmacological interventions [61]. Most patients experiencing de novo AHF typically exhibit reduced left ventricular ejection fraction (LVEF) [62]. However, even in cases where LVEF is preserved, cardiac damage tends to be reversible. De novo AHF often stems from two main events; pre-existing cardiomyopathy undergoes acute decompensation due to rhythm disturbances, infection, fluid imbalance, ischemia, or hypertension. Treatment approaches naturally differ depending on whether the event is primary or secondary.

Furthermore, the transition from de novo AHF to chronic heart failure (CHF) occurs relatively quickly after injury, with the complexity of activated molecular pathways influencing the specific disease trajectory [61]. Despite this, there is limited understanding of the therapeutic window and treatment targets to reverse HF or prevent CHF onset in de novo AHF patients, which could potentially enhance long-term outcomes [63]. The differing biology and molecular mechanisms based on etiology and comorbidities necessitate diverse approaches to mitigate the risk of progression towards chronic or advanced HF. In a recently published scientific statement by the European Society of Cardiology [64], the authors outline various pathways leading from acute heart failure (AHF) to chronic heart failure (CHF), stemming from the interplay among causative factors, genetic predisposition, environmental influences, and concurrent health conditions. Additionally, they explore potential molecular targets that could offer novel therapeutic avenues to enhance acute phase management and impede the progression towards CHF.

One of these targets could be endothelial dysfunction, which, as we have already seen, is characterized by dysregulated nitric oxide (NO) production, inflammation, and oxidative stress, which impair the vascular endothelium’s functions, such as the regulation of the vascular tone and the inflammatory processes [65]. These events are evident in conditions like sepsis, where endothelial dysfunction arises from various adaptive responses following decreased cardiac output, neurohumoral activation, vasoconstriction, increased oxidative stress, and NO imbalance. In sepsis, bacterial components primarily damage the endothelial barrier by activating toll-like receptors [66].

Additionally, acute inflammation affecting the coronary microvascular endothelium diminishes NO availability for nearby cardiomyocytes and disrupts cyclic guanosine monophosphate (cGMP)-protein kinase G signaling. This, coupled with reduced phosphorylation of the sarcomere protein titin, can increase left ventricular stiffness, exacerbating diastolic dysfunction and elevating the risk of AHF onset [39]. Diastolic dysfunction is commonly observed in hypertensive AHF patients, where acute fluid redistribution, NO insensitivity, and arterial/ventricular stiffening are critical determinants of the phenotype’s development [67].

The breakdown of the nitric oxide (NO)-soluble guanylyl cyclase (sGC)-cyclic guanosine monophosphate (cGMP) pathway is a common occurrence in individuals suffering from heart failure (HF) and results in endothelial dysfunction. This disruption is triggered by oxidative stress, which reduces the availability of NO and cGMP [68]. Increased levels of reactive oxygen species (ROS) can further lead to the oxidation of the soluble guanylyl cyclase (sGC) enzyme, making it unresponsive to NO stimulation and worsening endothelial dysfunction.

To address these issues, two innovative drug classes, sGC stimulators and sGC activators, have emerged as promising treatments for HF. Notably, the VICTORIA trial [69] evaluated the effectiveness and safety of Vericiguat, an oral sGC stimulator, in patients with reduced left ventricular ejection fraction (LVEF) and recently decompensated chronic heart failure (CHF).

Adrenomedullin (ADM), a vasoactive peptide elevated in individuals with volume overload and HF [70], plays a crucial role in vasodilation to maintain vascular integrity and reduce vascular leakage. Some studies [51,71,72,73,74,75,76,77] have shown that exogenous administration of ADM can lead to various benefits in HF, such as a reduction in the myocardial infarct size, cardiac myocyte apoptosis, left ventricular (LV) remodeling, and aldosterone levels, as well as an improvement in hemodynamics and survival.

Adrecizumab, a humanized monoclonal antibody targeting ADM, has shown promise and has improved clinical outcomes in acute decompensated heart failure by increasing plasma ADM concentrations and enhancing vascular integrity [70].

Similarly, ORM-3819, a calcium sensitizer/phosphodiesterase (PDE) inhibitor, induces endothelium-independent vasodilation and exerts positive inotropic effects by sensitizing cardiac troponin C (cTnC) to calcium. Animal studies [78,79] have demonstrated its potential to enhance contractile performance and alleviate coronary artery vasospasm.

Furthermore, serelaxin, a synthetic form of human relaxin-2, has been explored in patients with acute heart failure. Despite its diverse effects, including vasodilation and antifibrotic, angiogenic, antiapoptotic, and anti-inflammatory properties, serelaxin infusion did not reduce the incidence of death in patients with acute heart failure [80,81].

## 6. Clinical Trials and Endothelial Function

Several case–control studies have clearly documented a link between the progression of CVD and HF and endothelial dysfunction, highlighting how endothelial dysfunction could be a vital prognostic tool for evaluating CVD [16,19].

These correlations have strengthened the hypothesis that targeting endothelial dysfunction could lead to the amelioration of CVD. In recent clinical trials, the results have suggested that improving endothelial function may ameliorate cardiac biomechanics in patients with HF [82,83].

Two clinical trials reported how statins effectively improved endothelial function and hemodynamics in patients with CAD, atherosclerosis, and HF [84,85,86]. Antoniades et al. [86] found that statins, in patients with previous CAD, improved endothelial function, as assessed by FMD (flow-mediated dilatation). Additionally, they hypothesized a potential mechanism for this improvement: statins would increase vascular BH4 bioavailability via the up-regulation of guanosine triphosphate cyclohydrolase 1 and a reduction in endothelial NOS-derived superoxide, causing a rise in NO bioavailability.

The same group demonstrated that, in patients with ischemic HF, 4 weeks of treatment with atorvastatin improved endothelial function (measured through FMD and endothelial-independent vasodilation) and EPC mobilization and reduced tumor necrosis factor-α (TNF-α) [84].

In addition, Erbs et al. [85] found that, in 42 patients with HF, a high dose of rosuvastatin resulted in a dramatic improvement in FMD, LVEF, vascular endothelial growth factor (VEGF), and circulating EPCs and that these effects were accompanied by decreased levels of oxidized low-density lipoprotein.

Many other medications recommended for HFrEF have been further studied, yielding mixed results. Specifically, nebivolol, a b1-selective receptor blocker with vasodilating effects mediated by NO, demonstrated some efficacy in improving endothelial function ([87,88] while metoprolol, a b1-selective receptor blocker, and spironolactone, an aldosterone antagonist, had minimal to no effects [88].

Recently, in the ESC 2021 heart failure guidelines, SGLT2i and ARNI were recommended for the treatment of HFrEF [38].

There are two Italian studies that evaluate the effect of Sacubitril/Valsartan on endothelial dysfunction in patients with HFrEF. The first study showed improved endothelial function, left ventricular function, mitral regurgitation, and diastolic function in patients with dilated cardiomyopathy and reduced LVEF treated with Sacubitril/Valsartan, despite the small number of patients. However, it showed no effects on vascular stiffness [89]. In the second study, which included 100 patients, Cassano et al. [90] observed a significant improvement in arterial stiffness parameters and in endothelial function indices. The study demonstrated that a 6-month treatment with Sacubitril/Valsartan, in patients with HFrEF, improves endothelial dysfunction and arterial stiffness, by reducing oxidative stress, platelet activation, and circulating inflammatory biomarkers, without adverse effects.

Studies that assess SGLT2i and endothelial dysfunction are ongoing. Sposito et al. are assessing endothelial function with FMD in diabetic patients with CAD (ClinicalTrials.gov ID: NCT02919345).

There are many ongoing clinical trials assessing endothelial function in patients with CVD: nutraceutical therapies, sildenafil, sitagliptin, new oral anticoagulants, and albumin are only a few of the interventions currently under investigation.

More specifically, in the NUTRENDO (Effects of Neutraceutical Therapies on Endothelial Function, Platelet Aggregation, and Coronary Flow Reserve) trial (ClinicalTrials.gov Identifier: NCT02969070), the effect of nutraceutical therapies on endothelial function is being analyzed, specifically measuring the reactive hyperemia index in patients with hypercholesterolemia. Here, Trimarco et al. are also evaluating platelet aggregation and coronary flow reserve.

In their study, Liang et al. include patients with rheumatoid arthritis to understand whether sildenafil can improve endothelial function, as measured through FMD and biomarkers, and can therefore be an effective strategy for CVD prevention in a population associated with a twofold risk of CVD (ClinicalTrials.gov Identifier: NCT02908490).

The SAVORO (Sitagliptin Effects on Arterial Vasculature and Inflammation in Obesity; ClinicalTrials.gov Identifier: NCT02576288) trial is administering sitagliptin in patients with atherosclerosis, to see whether this treatment is able to improve endothelial function and carotid stiffness.

Kim et al. are testing the effect of new oral anticoagulants (dabigatran, rivaroxaban, warfarin) on endothelial dysfunction, as assessed via EndoPAT, and atherosclerosis prevention in patients with atrial fibrillation (ClinicalTrials.gov Identifier: NCT02544932).

Using EndoPAT, the Endothelial Function After Cardiac Surgery study (ClinicalTrials.gov Identifier: NCT02882074) is evaluating whether the administration of albumin during cardiac surgery could improve postoperative endothelial function compared to hydroxyethyl starch solutions.

In summary, a growing number of clinical trials support the hypothesis that drugs that effectively target endothelial dysfunction in patients with HF or CVD may be a new therapeutic option. However, many of these therapies have only been tested in a few clinical trials with a low number of included patients. There are ongoing trials assessing various types of interventions for treating endothelial dysfunction in patients with CVD, and at the same time, there are trials assessing the prognostic value of endothelial dysfunction for CVD progression.

## 7. Conclusions

Endothelial dysfunction is considered to be an emerging cardiovascular risk factor; as a result, it is the focus of many clinical investigations. The endothelial dysfunction pathophysiology plays a significant role in the development of CVD and in HF progression. Different methods for studying endothelial function have been validated and improved, and they have been shown to correlate with CVD outcomes. Nonetheless, more effective therapies targeting the endothelium to improve HF and CVD outcomes are needed. If endothelial function is recognized as a core component of CVD and HF, targeting endothelial dysfunction may represent a breakthrough in their treatment.

## Figures and Tables

**Table 1:** Characteristics of the methods for measuring endothelial function. FMD, flow-mediated vasodilation; IVUS, intravascular ultrasound; PAT, peripheral arterial tonometry.

Methods	Advantage	Disadvantage	Uncertain
**Quantitative Coronary Angiography ± IVUS**	Most direct Gold standard of early detection of endothelial dysfunction Heavily validated	Invasive procedure with connected risks Not feasible when screening large population Expensive Skilled technicians	None
**Flow-Mediated Vasodilation (FMD)**	Heavily validated Rapidity Noninvasive Easy to use Potentially feasible when screening large population	Expensive Skilled technicians	Many confounding factors due to ambience and diet (Food intake, drugs, vitamins, tobacco use...)
**Peripheral Arterial Tonometry (PAT)**	Operator independent Internal control Rapidity Noninvasive Easy to use Potentially feasible when screening large population Inexpensive	Microvascular measurement Unclear physiology	Correlation with FMD not clear Validity Does not measure endothelial disfunction directly

## Data Availability

Not applicable.
